# Supplementary material for: The Affect Misattribution in the Interpretation of Ambiguous Stimuli in Terms of Warmth vs. Competence: Behavioral Phenomenon and Its Neural Correlates
Source: Brain Sci. 2022 Aug 17;12(8):1093. doi: 10.3390/brainsci12081093 (PMC9406116; doi:10.3390/brainsci12081093)
Supplement: Supplementary file 1 [file brainsci-12-01093-s001.zip › supplementary File S2.pdf]

brain sciences

Supplementary File S2

A. The time course of the ERPs preceding the response

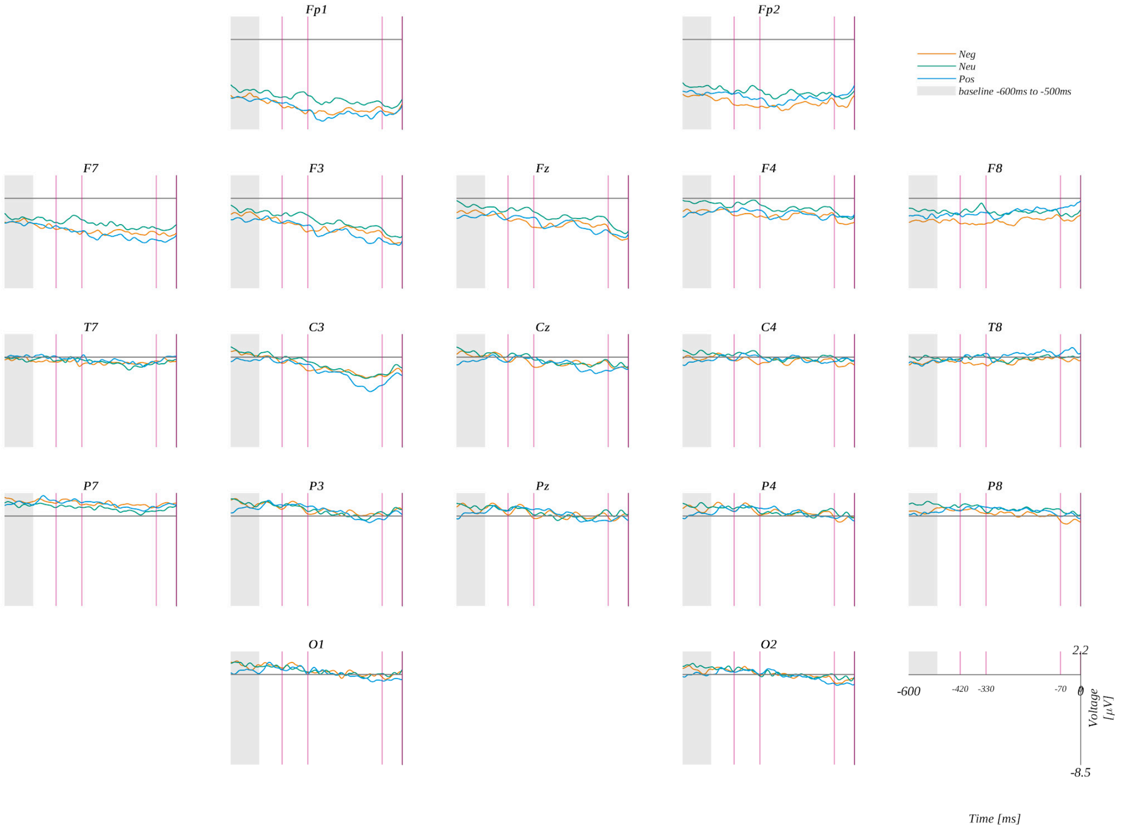

Figure S1. The time course of the ERP preceding the response (at time 0), for each level of valence, averaged across subjects without baseline correction. Gray rectangles indicate the time range selected for baseline corrections in the corresponding analysis.

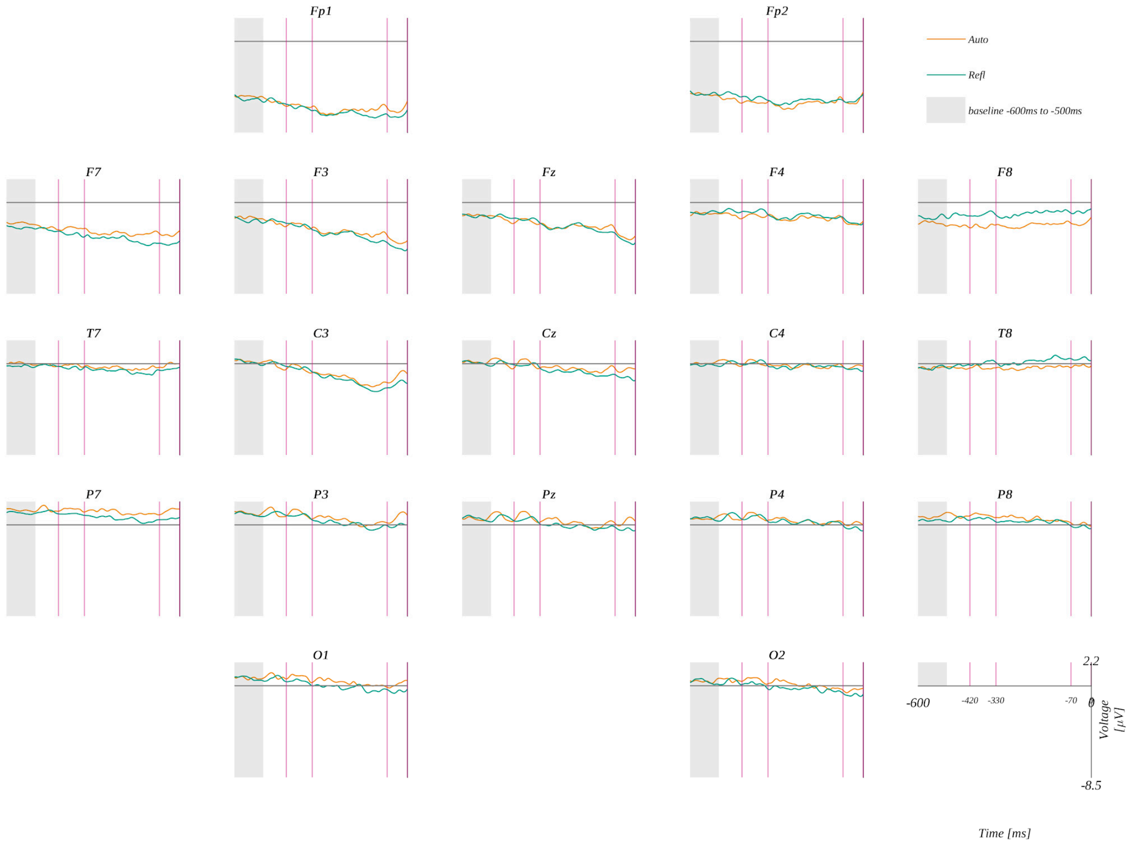

Figure S2. The time course of the ERP preceding the response (at time 0), for each level of origin, averaged across subjects without baseline correction. Gray rectangles indicate the time range selected for baseline corrections in the corresponding analysis.

B: Word reading and remembering task. ANOVA Table for the first level of analysis.

Time window: 60–145 ms

Effect	DFn	DFd	F	p
valence	2	54	1.36	0.266
origin	1	27	1.95	0.174
ROI	3	81	36.79	0.000
valence:origin	2	54	0.87	0.425
valence:ROI	6	162	0.33	0.919
origin:ROI	3	81	1.26	0.295
valence:origin:ROI	6	162	0.79	0.579

Time window: 145–235 ms

Effect	DFn	DFd	F	p
valence	2	54	1.65	0.202
origin	1	27	1.55	0.224
ROI	3	81	6.38	0.001
valence:origin	2	54	0.67	0.516
valence:ROI	6	162	0.08	0.998
origin:ROI	3	81	2.60	0.058
valence:origin:ROI	6	162	0.56	0.759

Time window: 235–350 ms

Effect	DFn	DFd	F	p
valence	2	54	0.76	0.472

origin	1	27	2.40	0.133
ROI	3	81	14.56	0.000
valence:origin	2	54	0.27	0.768
valence:ROI	6	162	0.56	0.763
origin:ROI	3	81	5.57	0.002
valence:origin:ROI	6	162	0.27	0.949

Time window: 350–500 ms

Effect	DFn	DFd	F	p
valence	2	54	2.33	0.107
origin	1	27	0.22	0.642
ROI	3	81	9.85	0.000
valence:origin	2	54	1.22	0.303
valence:ROI	6	162	0.37	0.897
origin:ROI	3	81	2.17	0.098
valence:origin:ROI	6	162	0.69	0.658

C: Stimulus locked effects for warmth-competence interpretation of octagram. ANOVA Table for first level of analysis.

Time window: 55–135 ms

Effect	DFn	DFd	F	p
valence	2	58	0.43	0.650
question	1	29	4.88	0.035
origin	1	29	0.36	0.553
ROI	3	87	18.19	0.000
valence:question	2	58	0.33	0.722
valence:origin	2	58	0.16	0.853
origin:question	1	29	4.62	0.040
valence:ROI	6	174	0.66	0.680
question:ROI	3	87	4.95	0.003
origin:ROI	3	87	0.15	0.932
valence:origin:question	2	58	0.25	0.776
valence:question:ROI	6	174	0.43	0.860
valence:origin:ROI	6	174	0.92	0.484
origin:question:ROI	3	87	2.54	0.062
valence:origin:question:ROI	6	174	2.21	0.044

Time window: 135–175 ms

Effect	DFn	DFd	F	p
valence	2	58	0.40	0.675
question	1	29	4.37	0.046
origin	1	29	4.75	0.038
ROI	3	87	2.49	0.066

valence:question	2	58	1.55	0.221
valence:origin	2	58	1.23	0.301
origin:question	1	29	0.07	0.793
valence:ROI	6	174	0.70	0.654
question:ROI	3	87	6.23	0.001
origin:ROI	3	87	0.19	0.904
valence:origin:question	2	58	0.12	0.884
valence:question:ROI	6	174	0.38	0.894
valence:origin:ROI	6	174	0.46	0.835
origin:question:ROI	3	87	2.42	0.072
valence:origin:question:ROI	6	174	1.16	0.330

Time window: 175–315 ms

Effect	DFn	DFd	F	p
valence	2	58	0.66	0.519
question	1	29	6.03	0.020
origin	1	29	6.45	0.017
ROI	3	87	27.85	0.000
valence:question	2	58	0.75	0.477
valence:origin	2	58	1.88	0.162
origin:question	1	29	0.16	0.693
valence:ROI	6	174	1.05	0.392
question:ROI	3	87	2.89	0.040
origin:ROI	3	87	1.42	0.243
valence:origin:question	2	58	0.13	0.883

valence:question:ROI	6	174	0.40	0.882
valence:origin:ROI	6	174	2.25	0.041
origin:question:ROI	3	87	2.04	0.114
valence:origin:question:ROI	6	174	1.20	0.309

Time window: 315–500 ms

Effect	DFn	DFd	F	p
valence	2	58	0.56	0.574
question	1	29	6.37	0.017
origin	1	29	8.19	0.008
ROI	3	87	44.41	0.000
valence:question	2	58	0.18	0.833
valence:origin	2	58	0.14	0.874
origin:question	1	29	0.35	0.560
valence:ROI	6	174	0.99	0.437
question:ROI	3	87	3.68	0.015
origin:ROI	3	87	1.16	0.328
valence:origin:question	2	58	0.23	0.793
valence:question:ROI	6	174	0.37	0.899
valence:origin:ROI	6	174	0.93	0.473
origin:question:ROI	3	87	2.56	0.060
valence:origin:question:ROI	6	174	0.82	0.555

D: Response locked effects for warmth-competence interpretation of octagrams. ANOVA Table for first level of analysis.

Time window: -420 ms to -330 ms

Effect	DFn	DFd	F	p
valence	2	50	2.32	0.109
question	1	25	0.45	0.507
origin	1	25	0.59	0.451
ROI	8	200	5.60	0.000
valence:question	2	50	6.48	0.003
valence:origin	2	50	1.17	0.320
origin:question	1	25	0.93	0.343
valence:ROI	16	400	2.18	0.005
question:ROI	8	200	0.60	0.777
origin:ROI	8	200	1.55	0.144
valence:origin:question	2	50	0.83	0.440
valence:question:ROI	16	400	1.64	0.056
valence:origin:ROI	16	400	1.54	0.084
origin:question:ROI	8	200	1.14	0.337
valence:origin:question:ROI	16	400	0.74	0.752

For the valence:ROI interaction Mauchly's test indicated that the assumption of sphericity had been violated ($\chi2(87)$ = 0.001 $p < .001$), therefore, degrees of freedom were corrected using Greenhouse-Geisser estimates of sphericity ($\varepsilon = .243$), and the Pr(>F[GG])=0.078. Therefore this effect is not significant.

Time window: -330 ms to -70 ms

Effect	DFn	DFd	F	p
valence	2	50	0.51	0.603

question	1	25	0.69	0.413
origin	1	25	1.03	0.319
ROI	8	200	7.99	0.000
valence:question	2	50	2.13	0.130
valence:origin	2	50	0.46	0.631
origin:question	1	25	0.20	0.659
valence:ROI	16	400	0.80	0.681
question:ROI	8	200	0.67	0.716
origin:ROI	8	200	0.65	0.739
valence:origin:question	2	50	0.88	0.421
valence:question:ROI	16	400	2.16	0.006
valence:origin:ROI	16	400	0.57	0.905
origin:question:ROI	8	200	0.48	0.869
valence:origin:question:ROI	16	400	0.38	0.987

Time window: -70 ms to 0 ms

Effect	DFn	DFd	F	p
valence	2	50	0.22	0.801
question	1	25	1.68	0.207
origin	1	25	1.68	0.207
ROI	8	200	10.58	0.000
valence:question	2	50	1.06	0.353
valence:origin	2	50	0.52	0.600
origin:question	1	25	0.49	0.489
valence:ROI	16	400	1.28	0.208
question:ROI	8	200	0.77	0.632

origin:ROI	8	200	1.25	0.273
valence:origin:question	2	50	3.48	0.039
valence:question:ROI	16	400	0.69	0.810
valence:origin:ROI	16	400	0.71	0.787
origin:question:ROI	8	200	0.94	0.483
valence:origin:question:ROI	16	400	0.61	0.881
